# Supplementary material for: Syndromic Surveillance Systems for Mass Gatherings: A Scoping Review
Source: Int J Environ Res Public Health. 2022 Apr 13;19(8):4673. doi: 10.3390/ijerph19084673 (PMC9026395; doi:10.3390/ijerph19084673)
Supplement: Supplementary file 1 [file ijerph-19-04673-s001.zip › Supplementary S1,S2.pdf]

Supplementary S1: Database queries

PubMed

(“mass gathering”[Title/Abstract] OR “large gathering”[Title/Abstract] OR "Anniversaries and Special Events"[Title/Abstract] OR "mass event"[Title/Abstract] OR "mass events"[Title/Abstract] OR "crowd"[Title/Abstract] OR "crowds"[Title/Abstract] OR "special event"[Title/Abstract] OR "sport event"[Title/Abstract] OR "sport events"[Title/Abstract] OR "sporting event"[Title/Abstract] OR "sporting events"[Title/Abstract] OR "music events"[Title/Abstract] OR "music event"[Title/Abstract] OR "concert"[Title/Abstract] OR "rave"[Title/Abstract] OR "raves"[Title/Abstract] OR "festival"[Title/Abstract] OR "festivals"[Title/Abstract] OR "occasion"[Title/Abstract] OR "occasions"[Title/Abstract] OR "social event"[Title/Abstract] OR "social events"[Title/Abstract])

AND

(“syndromic surveillance” [Title/Abstract] OR "syndromic diagnosis"[Title/Abstract] OR "syndromic diagnoses"[Title/Abstract] OR “syndromic” [Title/Abstract] OR “real-time surveillance” [Title/Abstract] OR "real time surveillance"[Title/Abstract])

Embase

(“mass gathering”:ab,ti OR “large gathering”:ab,ti OR "Anniversaries and Special Events":ab,ti OR "mass event":ab,ti OR "mass events":ab,ti OR "crowd":ab,ti OR "crowds":ab,ti OR "special event":ab,ti OR "sport event":ab,ti OR "sport events":ab,ti OR "sporting event":ab,ti OR "sporting events":ab,ti OR "music events":ab,ti OR "music event":ab,ti OR "concert":ab,ti OR "rave":ab,ti OR "raves":ab,ti OR "festival":ab,ti OR "festivals":ab,ti OR "occasion":ab,ti OR "occasions":ab,ti OR "social event":ab,ti OR "social events":ab,ti)

AND

(“syndromic surveillance”:ab,ti OR "syndromic diagnosis":ab,ti OR "syndromic diagnoses":ab,ti OR “syndromic”:ab,ti OR “real-time surveillance”:ab,ti OR "real time surveillance":ab,ti)

Web of Science

(TI = ((mass gathering OR large gathering OR anniversaries and special events OR mass event OR mass events OR crowd OR crowds OR special event OR sport event OR sport events OR sporting event OR sporting events OR music events OR music event OR

concert OR race OR raves OR festival OR festivals OR occasion OR occasions OR social event OR social events) AND (syndromic surveillance OR syndromic diagnosis OR syndromic diagnoses OR syndromic OR real-time surveillance OR real time surveillance))) OR (AB = ((mass gathering OR large gathering OR anniversaries and special events OR mass event OR mass events OR crowd OR crowds OR special event OR sport event OR sport events OR sporting event OR sporting events OR music events OR music event OR concert OR race OR raves OR festival OR festivals OR occasion OR occasions OR social event OR social events) AND (syndromic surveillance OR syndromic diagnosis OR syndromic diagnoses OR syndromic OR real-time surveillance OR real time surveillance)))

Supplementary S2–Inclusion/Exclusion Criteria

**Inclusion**	**Exclusion**
Use of syndromic surveillance for a mass gathering scenario	Either did not employ syndromic surveillance or was not used for a mass gathering
Articles in English	Non-English articles
Published during or after 2000	Published before 2000
